# Major Impact of Coping Styles on Anxiety and Depression Symptoms in Healthcare Workers During the Outbreak of COVID-19

**DOI:** 10.3389/fpsyg.2022.813295

**Published:** 2022-02-22

**Authors:** Dongke Wang, Jie Chen, Xinghuang Liu, Yan Jin, Yanling Ma, Xuelian Xiang, Ling Yang, Jun Song, Tao Bai, Xiaohua Hou

**Affiliations:** ^1^Division of Gastroenterology, Union Hospital, Tongji Medical College, Huazhong University of Science and Technology, Wuhan, China; ^2^Union Hospital, Tongji Medical College, Huazhong University of Science and Technology, Wuhan, China; ^3^Department of Respiratory and Critical Care Medicine, Union Hospital, Tongji Medical College, Huazhong University of Science and Technology, Wuhan, China

**Keywords:** COVID-19, healthcare workers, anxiety, depression, coping style

## Abstract

**Background:**

In the early days of COVID-19 outbreak, the normally orderly health system was severely challenged by large numbers of feverish patients and shortage of healthcare workers. The outbreak played a harmful role in the mental health of these healthcare workers.

**Objective:**

We aim to assess the prevalence of moderate or severe anxiety and depression symptoms (ADSs) of healthcare workers in different regions during COVID-19 disaster and identify the potential risk factors.

**Methods:**

We did a cross-sectional study on ADS of healthcare workers in epicenter-Hubei province and regions in lower epidemic-other provinces by questionnaire online. The data of ADS, the demographic characteristics, occupational exposure, physical condition, family situation, and coping styles were collected and analyzed.

**Results:**

A total of 24.68% of the respondents had experienced moderate or severe ADS. Moderate or severe ADSs were in a higher prevalence in Hubei (32.39%) than other provinces (18.22%). Suspicious symptoms on their own and in family members were independent risk factors of moderate or severe ADS of all health workers. Working on the frontline was the independent risk factor for participants in Hubei province, whereas quarantine was the independent risk factor for those in other provinces. Moreover, among all participants, those with negative coping style were more than four times more likely to have moderate or severe ADS than those with positive coping style.

**Conclusion:**

Moderate or severe ADSs were in a higher prevalence in healthcare workers of Hubei province during COVID-19 outbreak. The coping style may have major impact on ADS in such situation.

## Introduction

On 30 January 2020, given the severity of the outbreak, the World Health Organization (WHO) declared the coronavirus disease 2019 (COVID-19) as a Public Health Emergency of International Concern. By 24 o’clock on 10 February, the cumulative number of confirmed COVID-19 cases in China had reached 42,638, at the same time, Hubei province had 31,728 cumulative confirmed cases of COVID-19 according to the health commission^1,2^. The COVID-19 outbreak was unique in its rapidity of transmission and the large number of healthcare workers who were infected (China-WHO joint investigation report of novel coronavirus pneumonia). The normally orderly health system has been severely challenged by large numbers of feverish patients and shortage of healthcare workers. Great changes had taken place in the work and life of frontline healthcare workers. In addition, many frontline healthcare workers had more than 16 working hours per day on average (Huang and Zhao, 2020). Additionally, we had found that the increased workload was independently related to anxiety, depression, and job burnout among healthcare workers in the previous study (Chen et al., 2020; Liu et al., 2020). Worse still, they were great risk group for infection by the virus. This biodisaster as a life-threatening and life-altering event played a harmful role on the mental health of these healthcare workers.

Coping was defined as thoughts and behaviors that people use to manage the internal and external demands of situations that are appraised as stressful (Folkman and Lazarus, 1980). The earliest classification distinguished coping into problem-focused coping and emotion-focused coping, which were often fitted by later conceptualizations of coping, and also the concepts of positive coping and negative coping. In the majority of studies of coping and adjustment, negative coping has been associated with higher levels of distress (Folkman and Moskowitz, 2004). But it is still unknown whether the coping styles can have an important impact on how healthcare workers respond to this biodisaster and psychological distress. We hypotheses that coping styles and factors related to COVID-19 disaster may potentially contribute to anxiety and depression of healthcare workers in different provinces of China. Therefore, we aim to assess the psychological impact of COVID-19 disaster on Chinese healthcare workers and identify the possible risk factors, especially coping style, associated with anxiety and depression symptoms (ADSs) by a web-based cross-sectional study, to offer favorable conditions in times of extreme distress, such as the current and future pandemics.

## Materials and Methods

### Study Design and Participants

From 9 February 2020 to 13 February 2020, we did a cross-sectional study of the epidemiology of ADSs of healthcare workers in China. Healthcare workers in hospitals both in epicenter-Hubei province and regions in low epidemic-other provinces who were still at work during the outbreak of COVID-19 were eligible in this survey. Healthcare workers were all local staffs except for support staffs from other regions during this outbreak. The study was conducted by convenience sampling online. The purpose, meaning, and confidentiality of personal information of the research were explained before eligible healthcare workers filled out the self-reported questionnaire. Healthcare workers were recruited when they gave their permission to participate and then voluntarily completed the questionnaire in WeChat small program^3^ after scanning the specific two-dimensional code. Ethics approval was received from the research Ethics Committees of the Wuhan Union Hospital, Tongji Medical College, Huazhong University of Science and Technology.

The demographic characteristics, occupational exposure, physical condition, and family situation were collected in this questionnaire. The generalized anxiety disorder 7-item scale (GAD-7), 9-item Patient Health Questionnaire (PHQ-9), and the 20-item Trait Coping Style Questionnaire (TCSQ) were used to measure psychological status and coping styles (He et al., 2010; Wang et al., 2014; Zhou et al., 2016).

### Basic Characteristics

The demographic characteristics include age, sex (male and female), occupation (doctor, nurse, and other healthcare workers), and title of occupation (primary title, senior title, and none). Primary title included medic, resident doctor, attending doctor, primary nurse, and primary nurse practitioner; senior title included associate chief physician or associate professor, chief physician or professor, nurse-in-charge, deputy chief nurse, and senior nurse. The occupational exposure characteristics included location (Hubei province and other provinces) and working environment (frontline and non-frontline). Hubei province was the epicenter of COVID-19 outbreak, whereas other provinces in China mainland were in lower epidemic. “Frontline” means that healthcare workers directly contacted with patients with COVID-19. Other healthcare workers in hospital were defined as “non-frontline.”

Physical condition and family situation were collected by questions answering “yes” or “no.” Physical condition was evaluated by “Did you have suspicious symptoms of COVID-19 (fever, shortness of breath, cough, sputum, fatigue, muscular soreness, diarrhea, nausea, and vomiting) in last 2 weeks?” Healthcare workers who had one of the suspicious symptoms can choose “yes.” The family situation was assessed by the following questions: (1) Are there the old or the child in your family needing to be taken care of? (2) Are you quarantined from your family members? (3) Did your family members have suspicious symptoms in last 2 weeks? Participants who answered “yes” in “question (1)” were defined as family caregivers. Suspicious symptoms in family members were consistent with those in their own physical condition.

### Measurement of Anxiety and Depression Symptoms

The degree of ADSs was assessed by GAD-7 and PHQ-9, respectively. There were seven items, each item ranges from 0 to 3, and the total score of GAD-7 can be divided into four severity levels: severe (>14), moderate (10–14), mild (5–9), and no (≤4) anxiety (Yang et al., 2019). Chinese version of GAD-7 was employed in this study, which has good reliability and validity in Chinese population, with Cronbach’s alpha coefficient at 0.898 and Kappa value at 0.825 (He et al., 2010; Yang et al., 2019). Additionally, a cutoff score of 10 was recommended for none or mild anxiety and moderate or severe anxiety distinction (He et al., 2010; Kroenke et al., 2010; Tong et al., 2016). There were nine items with each item ranging from 0 to 3. According to the total score, the severity of depression is defined as follows: severe (>20), moderate to severe (15–20), moderate (10–14), mild (5–9), and no (≤4) depression (Wang et al., 2014; Yang et al., 2019). The Chinese version of PHQ-9 has also been proved to have high reliability and validity in general Chinese population, whose Cronbach’s alpha equals to 0.86 (Wang et al., 2014; Du et al., 2017). Additionally, a total score larger than 9 was recommended as cutoff score to distinguish the none or mild and the moderate or severe depression (Kroenke et al., 2010; Wang et al., 2014; Du et al., 2017). In the analysis, participants who had moderate or severe anxiety or depression symptoms were combined into participants with moderate or severe ADS.

### Measurement of Coping Style

The 20-item TCSQ was employed in this study to evaluate the coping style positive coping (PC) or negative coping (NC) of participants. There are 10 items for each coping style, with each item scores from 1 to 5. The Chinese version of TCSQ was proved to be valid and reliable in Chinese population, with Cronbach’s alpha for each coping style dimension at 0.790 and 0.776, respectively (Ding et al., 2015; Qiao et al., 2016). Individuals are more likely to use the coping style, if its score is higher than that of another coping style (Ding et al., 2015; Zhou et al., 2016).

### Statistical Analysis

Statistical difference in categorical variable was detected by chi-square (χ^2^) test. Student’s *t*-test was performed to detect statistical significance in continuous variables. Step-by-step multiple logistic regression was conducted to find independent risk factors for moderate and severe ADS and also calculate odds ratios (ORs) with adjusted for demographic characteristics, such as sex, age, occupation, and so on. The area under the receiver operating characteristic (ROC) curve, also known as C statistic, was employed to evaluate clinical usefulness of the regression model with a cutoff value of 0.7 (Meurer and Tolles, 2017). We used R 3.6.0^4^ to perform all the statistical analyses with a significant double-tailed *p*-value of less than 0.05.

## Results

### Basic Characteristics

**FIGURE 1:**
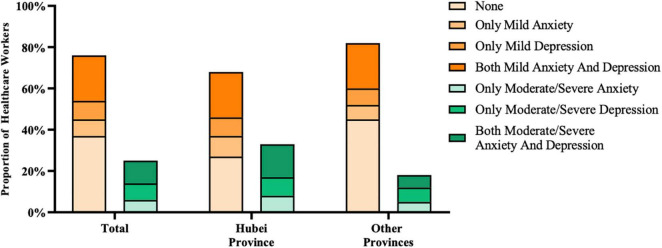
Distribution of ADSs in Hubei province and other provinces. The occurrence of ADSs can be single or coexistent, although most participants in our study had both ADSs. Healthcare workers in Hubei province had a higher prevalence of moderate or severe ADS than those of other provinces.

Totally, 928 effective questionnaires were recovered online (Table 1). A total of 60.78% (564/928) of them were doctors, 34.05% (316/928) were nurses, and 5.17% (48/928) were other healthcare workers. A total of 45.58% (423/928) of them came from Hubei Province, which was hardest hit by COVID-19, and 54.42% (505/928) were from other provinces in China mainland. Almost a quarter of healthcare workers (24.68%, 229/928) experienced moderate or severe ADS, and 137 of them came from Hubei province. Moderate or severe ADSs were in a higher prevalence (*p* < 0.001) in Hubei (32.39%, 137/423) than other provinces (18.22%, 92/505). Coexistence of ADSs was more common (*p* < 0.001) in Hubei province (15.60%, 66/423) than other provinces (6.34%, 32/505) (Figure 1). Additionally, the detailed numbers of participants with different anxiety and depression status in Hubei and other provinces are contained in Supplementary Table 1.

**TABLE 1:** General characteristics of participants according to ADSs (*n* = 928).

Variables	Total	None/mild anxiety and depression (%)	Moderate/severe anxiety and depression (%)	Test value (χ^2^/t)	*p*-value
**Demographic characteristic**					
**Age**	/	36.50 ± 8.57	36.59 ± 8.37	–0.135	0.893
**Sex**				1.060	0.303
Male	295	229 (77.63)	66 (22.37)		
Female	633	470 (74.25)	163 (25.75)		
**Occupation**				1.019	0.601
Doctor	564	422 (74.82)	142 (25.18)		
Nurse	316	243 (76.90)	73 (23.10)		
Others	48	34 (70.83)	14 (29.17)		
**Titles of occupation**				3.236	0.198
Primary title	552	407 (73.73)	145 (26.27)		
Senior title	328	258 (78.66)	70 (21.34)		
None	48	34 (70.83)	14 (29.17)		
**Occupational exposure characteristic**					
**Location**				24.109	< 0.001***
Hubei province	423	286 (67.61)	137 (32.39)		
Other provinces	505	413 (81.78)	92 (18.22)		
**Working environment**				15.059	< 0.001***
Frontline	258	171 (66.28)	87 (33.72)		
Non-frontline	670	528 (78.81)	142 (21.19)		
**Physical condition**					
**Having** **suspicious symptoms related to COVID-19 in last 2 weeks**				45.516	< 0.001***
Yes	347	218 (62.82)	129 (37.18)		
No	581	481 (82.79)	100 (17.21)		
**Family situation**					
**Being family caregivers of the old or the child**				4.976	0.026*
Yes	585	426 (72.82)	159 (27.18)		
No	343	273 (79.59)	70 (20.41)		
**Being quarantined from family**				14.762	< 0.001***
Yes	212	138 (65.09)	74 (34.91)		
No	716	561 (78.35)	155 (21.65)		
**Having suspicious symptoms related to COVID-19 in family members in last 2 weeks**				16.267	< 0.001***
Yes	95	55 (57.89)	40 (42.11)		
No	833	644 (77.31)	189 (22.69)		
**Coping styles**					
**Coping style assessed by Trait Coping Style Questionnaire**				93.734	< 0.001***
Negative coping style	290	159 (54.83)	131 (45.17)		
Positive coping style	638	540 (84.64)	98 (15.36)		

Values were expressed as n (%). *p < 0.05, ***p < 0.001.

### Related Risk Factors of Anxiety and Depression Symptoms Across China

In the univariate analysis, all of the occupational exposure characteristic, physical condition, family situation, and coping styles had effects on moderate or severe ADSs (Table 1). In the step-by-step logistical analysis, multiple factors can independently influence the occurrence of moderate or severe ADSs (Table 2). The C statistic of the best regression model of the whole participants in China was 0.752. Participants with the location of Hubei province (OR 1.600, 95% CI: 1.116–2.294), suspicious symptoms in last 2 weeks (OR 2.254, 95% CI: 1.612–3.157), the suspicious symptoms of family members in last 2 weeks (OR 1.776, 95% CI: 1.083–2.890), and negative coping style (OR 4.350, 95% CI: 3.134–6.065) were more likely to have moderate or severe ADS than those without the exposures.

**TABLE 2:** Multiple logistic regression analysis with risk factors for moderate/severe ADSs (*n* = 928) (C statistic = 0.752).

Variables	*p*-value	OR (95% CI)
**Occupational exposure situation**		
**Location**		
Hubei province	0.011*	1.600 (1.116, 2.294)
Other provinces		1
**Work environment**		
Frontline	0.062	1.431 (0.980, 2.084)
Non-frontline		1
**Physical condition**		
**Having suspicious symptoms related to COVID-19 in last 2 weeks**		
Yes	< 0.001***	2.254 (1.612, 3.157)
No		1
**Family situation**		
**Being family caregivers of the old or the child**		
Yes	0.080	1.370 (0.967, 1.954)
No		1
**Having suspicious symptoms related to COVID-19 in family members in last 2 weeks**		
Yes	0.022*	1.776 (1.083, 2.890)
No		1
**Coping style**		
**Coping style assessed by Trait Coping Style Questionnaire**		
Negative coping style	< 0.001***	4.350 (3.134, 6.065)
Positive coping style		1

*p < 0.05, ***p < 0.001.

### Major Impact of Coping Styles on Anxiety and Depression Symptom

About 31.25% (290/928) of healthcare workers had negative coping style and 68.75% (638/928) had positive coping style. Participants with negative coping style were more than four times more likely to have moderate or severe ADS than those with positive coping style, in both Hubei province and other provinces (Table 3). Therefore, negative coping style may have major impact on ADS both in Hubei and other provinces. The detailed prevalence and severity of AD0053 in groups categorized by coping styles and location can be seen in Supplementary Tables 2–4, which were in line with the result of the multiple logistic regressions.

**TABLE 3:** Two separate multiple logistic regression analysis with risk factors for ADSs in Hubei province (C statistic = 0.720) and other provinces (C statistic = 0.765).

Variables	Hubei province (*n* = 423)		Other provinces (*n* = 505)	
	*p*-Value	OR (95% CI)	*p*-Value	OR (95% CI)
**Occupational exposure characteristic**				
**Work environment**				
Frontline	0.020*	1.704 (1.092, 2.679)	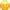	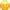
Non-frontline		1	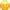	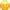
**Physical condition**				
**Having suspicious symptoms related to COVID-19 in last 2 weeks**				
Yes	0.004**	1.926 (1.229, 3.035)	<0.001***	2.649 (1.600, 4.390)
No		1		1
**Family situation**				
**Having suspicious symptoms related to COVID-19 in family members in last 2 weeks**				
Yes	0.051	1.918 (0.995, 3.697)	0.073	1.963 (0.917, 4.054)
No		1		1
**Being quarantined from family members**				
Yes	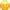	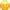	0.002**	3.381 (1.519, 7.420)
No	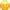	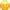		1
**Coping style**				
**Coping style assessed by Trait Coping Style Questionnaire**				
Negative coping style	<0.001***	4.309 (2.760, 6.798)	<0.001***	4.565 (2.792, 7.536)
Positive coping style		1		1

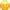
 This factor was not included in the optimization model in the multiple logistic regression analysis in its group of specific location (Hubei province or other provinces). For example, work environment was not included in the optimization model for risk factors of ADS in other provinces. *p < 0.05, **p < 0.01, ***p < 0.001.

### Different Related Risk Factors of Anxiety and Depression Symptom of Hubei Province and Other Provinces

**FIGURE 2:**
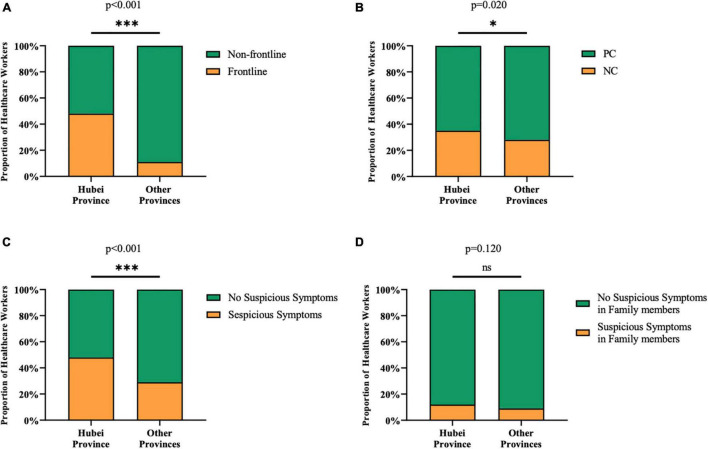
Difference in potential risk factors on ADSs between Hubei province and other provinces. **(A)** More healthcare workers worked on the frontline in Hubei province (48%) than those in other provinces (11%). **(B)** The proportion of participants with negative coping style was slightly larger in Hubei (35%) than that of other provinces (28%). **(C)** Healthcare workers in Hubei province (48%) were more frequent to have suspicious symptoms compared with other provinces (29%). **(D)** There was no significant difference in the proportion of healthcare workers who had family members with suspicious symptoms between Hubei (12%) and other provinces (9%). *p < 0.05, ^***^p < 0.001, ^ns^*p* > 0.05.

According to Figure 2, the proportion of the frontline (*p* < 0.001), suspicious symptoms (*p* < 0.001) on their own of healthcare workers in Hubei province were significantly higher than those in other provinces. On the other hand, negative coping style was more common in Hubei province (*p* = 0.02).

Separate logistic regression analysis was done for different locations. The C statistic of Hubei province and other provinces was 0.720 and 0.765, respectively. Among the 11 variables, only three of them working on the frontline (OR 1.704, 95% CI: 1.092–2.679), suspicious symptoms (OR 1.926, 95% CI: 1.229–3.035) in last 2 weeks and negative coping style (OR 4.309, 95% CI: 2.760-6.798) were the independent factors for healthcare workers in Hubei province (Table 3). However, in other provinces, suspicious symptoms in last 2 weeks (OR 2.649, 95% CI: 1.600–4.390), quarantine (OR 3.381, 95% CI: 1.519–7.420), and negative coping style (OR 4.565, 95% CI: 2.792–7.536) were independent factors (Table 3). Suspicious symptoms in family members in last 2 weeks may be the potential factors to healthcare workers in Hubei and other provinces.

## Discussion

We found that 24.68% of 928 healthcare workers suffered moderate or severe ADS during the outbreak of COVID-19. Coping style increased the risk of ADS to a great extent. Suspicious symptoms on their own or in family members in last 2 weeks also had independent effect on moderate or severe ADS among all healthcare workers. However, working on the frontline was the independent risk factor for participants in Hubei province, whereas quarantine was an independent risk factor for those in other provinces.

Healthcare workers with both of ADSs were more than those with the single. The two sets of symptoms were different, but researchers have found that many showed a mixed anxious-depressed symptom picture that cannot be characterized as one type of disorder or the other (Downing, 1974). Thus, the two were perhaps mutually reinforcing each other (Davis et al., 2010). For example, the accumulation of stressful work experiences of COVID-19 plausibly underlined the development of depression following anxiety, and the sad mood induction of depression caused by the spreading and worsening of COVID-19 may sensitize and facilitate the anxiety. Mental disorder screening of healthcare workers should address both anxiety and depression to discover problems.

Hubei province was in a higher prevalence of moderate or severe ADS. Hubei province was the epicenter of the outbreak of COVID-19, and it is where healthcare workers fight the biodisaster first in China. During the outbreak of SARS in 2003–2004, Hong Kong was more seriously hit by SARS than other countries, such as Singapore and Canada. As reported in different studies, mental disorders assessed by General Health Questionnaire (GHQ) were more common in Hong Kong rather than other countries (Chan and Huak, 2004; Nickell et al., 2004; Tam et al., 2004). But the comparison among these results was lack of rigor, because of the various survey times and the different ways of data collection and analysis. However, the differences in mental symptoms in the epicenter and other regions were compared in our study during the biodisaster. More adverse, mental symptoms in Hubei province can partly be explained by the higher proportion of the frontline, more suspicious symptoms on their own and more negative coping styles. In the current phrase, online psychological assistance specially for healthcare workers, such as remotely delivered psychological therapies, chat lines, and psycho-education, should be provided in time.

Healthcare workers with suspicious symptoms, which were reported as the initial symptoms of SARS-CoV-2 infection (Guan et al., 2020), were more likely to have moderate or severe ADS. An intriguing study suggested that dyspnea one of the usual suspicious symptoms of COVID-19 can reflect an underlying anxiety disorder and there were pathophysiologic relationships between them (Smoller et al., 1996). On the contrary, suspicious symptoms would definitely increase the fear of illness and death, so we think that the scientific research on COVID-19 should be speeded to fight this disease, and necessary protective equipment for healthcare workers should be paid sufficient attention to decrease the incidence of infection.

Suspicious symptoms in family members and family members needing to be taken care of were also independent factors. Uncertainty about the physical health of the family, fear of losing the loved ones, and the decreased family support may contribute to the ADS among participants with poor family situation (Köse et al., 2016). Therefore, the family situation during this biodisaster should not be ignored in the development of ADS. It has been also proved that family caregivers reported increased psychosocial outcomes, so the challenges of caregiving and heavy workload in healthcare workers were exacerbated by the COVID-19 pandemic (Chen et al., 2020; Beach et al., 2021). Increased multifaceted support should be provided during this epidemic, such as facilitating access to telehealth support services for healthcare workers and accessible, affordable, and timely medical care for their family members.

More participants on the frontline (33.72%) had moderate or severe ADS than the non-frontline (21.19%), but the frontline was an independent risk factor in Hubei province not in others. In the epicenter-Hubei province, the workload of healthcare workers may be harder than those in other provinces. During the harder situation, the change in work environment, a lack of professionalism of emerging COVID-19, and the elevated risk of infection of the healthcare workers on the frontline may make them more adverse emotional reactions. Healthcare workers being quarantined from family members had over three times the odds of moderate or severe ADS in other provinces, but it was not the independent factor in Hubei province. The quarantine might be more common in the epicenter than others, so there might be more colleagues standing together and facing all the difficulties. In other provinces, being quarantined was rare, so healthcare workers may be more inundated with the fear of death, the loss of social supports, the shame of SARS-CoV-2 infection, and even feel like discriminated by the society (Im et al., 2018). Therefore, the regional differences should be taken into account when policymaking on mental health of healthcare workers.

According to the result of multiple logistic regression analysis, coping style had a major impact on the occurrence of moderate or severe ADS both in Hubei and other provinces. A person who applied positive coping may regard stress as opportunities and challenges and then seek solutions to the emerging problems. On the contrary, participants with negative coping are more likely to focus on the negative emotions related to stress event rather than the solution of problem. As demonstrated in previous studies, negative coping would turn to high levels of emotional exhaustion, and positive coping was a positive source to fight against the depressed mood (Ding et al., 2015; Segerstrom and Smith, 2019). Therefore, healthcare workers with negative coping styles referred to negative emotions (such as ADS) when exposing to the stressor of this outbreak. Healthcare administrators should give special attention to coping styles of healthcare workers, and regard them as an important part of promoting the mental health of themselves. Some active interventions have been proved to be effective in the previous studies, including that foster connections of the team, provide education and training to develop active knowledge, skills, attitudes and behaviors, and learn from experiences of other biodisasters (e.g., SARS). Individual strategies and organizational support are both integral to equipping individuals to fight with work-related challenges (Phua et al., 2005; Gillman et al., 2015).

We hope that our findings may also make sense for the future public health crises. Healthcare workers always protect patients from any public health crisis, but the challenges of a public health crisis will inevitably spill over to themselves. At the same time dealing with their own physical health, mental health should also be emphasized. Targeted policy recommendations based on findings from researches could be implemented as soon as possible to avoid serious anxiety or depression disorders of healthcare workers. Besides, there are some limitations within this cross-sectional study. First of all, the only conclusion can be drawn is that those factors discussed above are possible risk factors related to ADS, since the cross-sectional study is not able to explain the relationship between causes and effects. Second, selective bias cannot be avoided when applying convenience sampling by online questionnaires. Even so, the results of our study may provide evidence for further psychological interventions and health policies. The psychological distress of healthcare workers during or post prevalence of COVID-19 still needs deep investigation.

## Conclusion

During the outbreak of COVID-19, moderate or severe ADSs were in a higher occurrence rate in healthcare workers in the epicenter-Hubei, China. Occupation exposure, physical condition, family situation, and coping style may have independent effects on ADS, but coping style may have a major impact on ADS.

## Data Availability Statement

The raw data supporting the conclusions of this article will be made available by the authors, without undue reservation.

## Ethics Statement

The studies involving human participants were reviewed and approved by the Research Ethics Committees of Union Hospital, Tongji Medical College, Huazhong University of Science and Technology. Written informed consent for participation was not required for this study in accordance with the National Legislation and Institutional Requirements.

## Author Contributions

DW, JC, and XL designed the study. YJ and YM collected the data. LY and XX undertook the statistical analysis. DW and JC wrote the first draft of the manuscript. XL, TB, and JS revised the manuscript. TB and XH supervised the study. All authors actively participated in the study and reviewed and approved the final manuscript.

## Conflict of Interest

The authors declare that the research was conducted in the absence of any commercial or financial relationships that could be construed as a potential conflict of interest.

## Publisher’s Note

All claims expressed in this article are solely those of the authors and do not necessarily represent those of their affiliated organizations, or those of the publisher, the editors and the reviewers. Any product that may be evaluated in this article, or claim that may be made by its manufacturer, is not guaranteed or endorsed by the publisher.

## Footnotes

1
http://www.nhc.gov.cn

2
http://wjw.hubei.gov.cn

3
https://web.wechat.com/

4
https://www.r-project.org/
