# Supplementary material for: Major Impact of Coping Styles on Anxiety and Depression Symptoms in Healthcare Workers During the Outbreak of COVID-19
Source: Front Psychol. 2022 Feb 22;13:813295. doi: 10.3389/fpsyg.2022.813295 (PMC8902159; doi:10.3389/fpsyg.2022.813295)
Supplement: Supplementary file 1 [file Table_1.docx]

**Supplementary Table 1. Distribution of Participants Based on Assessment of Anxiety and Depression Symptoms**

| **Anxiety and Depression Symptoms** | **Total Participants^1^ (n=928)** | **Hubei Province**^2^ **(n=423)** | **Other Provinces^2^ (n=505)** | **P value^3^** |
| --- | --- | --- | --- | --- |
| **None/Mild Anxiety and Depression Symptoms** | 699 (75.32) | 286 (67.61) | 413 (81.78) | **<0.001 ***** |
| None Anxiety or Depression | 340 (36.64) | 114 (26.95) | 226 (44.75) | **<0.001 ***** |
| Only Mild Anxiety | 78 (8.41) | 41 (9.69) | 37 (7.33) | 0.240 |
| Only Mild Depression | 80 (8.62) | 40 (9.46) | 40 (7.92) | 0.476 |
| Both Mild Anxiety and Depression | 201 (21.66) | 91 (21.51) | 110 (21.78) | 0.985 |
| **Moderate/Severe Anxiety and Depression Symptoms** | 229 (24.68) | 137 (32.39) | 92 (18.22) | **<0.001 ***** |
| Only Moderate/Severe Anxiety | 57 (6.14) | 32 (7.57) | 25 (4.95) | 0.130 |
| Only Moderate/Severe Depression | 74 (7.97) | 39 (9.22) | 35 (6.93) | 0.246 |
| Both Moderate/Severe Anxiety and Depression | 98 (10.56) | 66 (15.60) | 32 (6.34) | **<0.001 ***** |

1. Values in this column were expressed as the number (n) of total participants in different symptoms groups and its proportion (%) of the total (n=928).
2. Values in this column were expressed as the number (n) of each group and its proportion (%) of each region (n=423/505).
3. P value for proportions of Hubei province and other provinces of each line.

**Supplementary Table 2. Prevalence of Moderate/Severe Anxiety or Depression Symptoms in Hubei and Other Provinces**

| **Variables** | **NC** | | **P value^2^** | **PC** | | **P value^2^** |
| --- | --- | --- | --- | --- | --- | --- |
|  | Hubei Province | Other Provinces |  | Hubei Province | Other Provinces |  |
| **Work environment** | | | | | | |
| Frontline | 60.61%^1^ | 47.37% | 0.44 | 25.36% | 8.57% | 0.056 |
| Non-frontline | 46.99% | 35.25% | 0.12 | 16.91% | 11.25% | 0.13 |
| **P value^3^** | 0.14 | 0.45 | / | 0.12 | 0.78 | / |
| **Suspicious symptoms** | | | | | | |
| Yes | 65.38% | 52.94% | 0.22 | 25.81% | 20.21% | 0.42 |
| No | 39.44% | 27.78% | 0.16 | 17.33% | 7.78% | **0.0049 **** |
| **P value^3^** | **0.0027**** | **0.0052**** | / | 0.12 | **0.0018**** | / |
| **Suspicious symptoms in family members** | | | | | | |
| Yes | 54.46% | 36.89% | **0.014 *** | 20.87% | 14.05% | 0.075 |
| No | 48.65% | 36.84% | 0.42 | 22.06% | 4.92% | **<0.001 ***** |
| **P value^3^** | 0.67 | 1 | / | 0.97 | **0.014*** | / |

1. Values in this table were expressed as the proportion of moderate/severe anxiety and depression symptoms for their own groups.

For example, 60.61% (40/66) of participants with negative coping style who were both in Hubei province and on the frontline had moderate/severe anxiety and depression symptoms.

1. P value for proportions of Hubei province and other provinces in each line.
2. P value for proportion of frontline and non-frontline, suspicious symptoms or not, suspicious symptoms in family members or not in each column.

**Supplementary Table 3 The severity of Anxiety Symptoms in different coping styles**

| **Variables** | **NC** | | **P value^2^** | **PC** | | **P value^2^** |
| --- | --- | --- | --- | --- | --- | --- |
|  | Hubei Province | Other Provinces |  | Hubei Province | Other Provinces |  |
| **Work environment** | | | | | | |
| Frontline | 8.5 (7-14.75)^1^ | 7 (4-10) | 0.04 * | 5 (2-8) | 3 (1-6) | **0.006 *** |
| Non-frontline | 7 (5.5-11) | 7 (4.25-9) | 0.11 | 4 (2-7) | 3 (0-6) | **0.0012 **** |
| **P value^3^** | **0.026*** | 0.81 | / | 0.092 | 0.88 | / |
| **Suspected symptoms** | | | | | | |
| Yes | 10 (7-14) | 7 (6-11) | 0.015 * | 5.5 (2-8) | 4 (2-6.75) | **0.042 *** |
| No | 7 (6-8) | 7 (2-8) | 0.17 | 4 (2-7) | 2 (0-5) | **<0.001 ***** |
| **P value^3^** | **<0.001***** | **0.036*** | / | **0.022*** | <0.001*** | / |
| **Suspected symptoms in family members** | | | | | | |
| Yes | 7 (6-13) | 7 (5-9.5) | 0.012 * | 5 (2-7) | 3 (1-6) | **<0.001 ***** |
| No | 7 (6-12) | 7 (3-7) |  | 3 (1-7) | 2 (0-5) | **0.022*** |
| **P value^3^** | 0.85 | 0.36 | / | **0.0086*** | **0.0034**** | / |

1. Values were expressed as median (25^th^ percentile - 75^th^ percentile) of anxiety score for their own groups

For example, the median (25th percentile - 75th percentile) score of anxiety symptoms in participants with negative coping style who were both in Hubei province and on the frontline was 8.5 (7-14.75).

1. P value for scores of Hubei province and other provinces in each line.
2. P value for scores of frontline and non-frontline, suspicious symptoms or not, suspicious symptoms in family members or not in each column.

**Supplementary Table 4 The severity of Depression Symptoms in different coping styles**

| **Variables** | **NC** | | **P value^2^** | **PC** | | **P value^2^** |
| --- | --- | --- | --- | --- | --- | --- |
|  | Hubei Province | Other Provinces |  | Hubei Province | Other Provinces |  |
| **Work environment** | | | | | | |
| Frontline | 10 (5.25-13)^1^ | 7 (4.5-10) | 0.067 | 5 (3-8.75) | 3 (1-6) | **0.008 **** |
| Non-frontline | 8 (5-12) | 8 (5-10) | 0.28 | 4.5 (2-7) | 3 (1-6) | **0.003 **** |
| **P value^3^** | 0.18 | 0.73 | / | 0.056 | 0.86 | / |
| **Suspected symptoms** | | | | | | |
| Yes | 11 (7-14) | 8 (5-12.5) | 0.053 | 6 (3-8) | 5 (2.25-8) | 0.11 |
| No | 7 (4-10) | 7.5 (4-9) | 0.65 | 4 (2-7) | 3 (0-6) | **<0.001***** |
| **P value^3^** | **<0.001***** | **0.04*** | **/** | **0.001**** | **<0.001***** | / |
| **Suspected symptoms in family members** | | | | | | |
| Yes | 9 (5-13) | 8 (5-10) | 0.069 | 5 (3-8) | 3 (1-6) | **<0.001 ***** |
| No | 9 (6-11) | 8 (3.25-10.75) | 0.31 | 3 (1.75-6.25) | 3 (0.25-6) | 0.15 |
| **P value^3^** | 0.95 | 0.98 | / | **0.0056**** | 0.21 | / |

1. Values were expressed as median (25^th^ percentile - 75^th^ percentile) of depression score for their own groups.

For example, the median (25th percentile - 75th percentile) score of depression symptoms in participants with negative coping style who were both in Hubei province and on the frontline was 10 (5.25-13).

1. P value for scores of Hubei province and other provinces in each line.
2. P value for scores of frontline and non-frontline, suspicious symptoms or not, suspicious symptoms in family members or not in each column.
